# Melatonin and Ischemic Stroke: Mechanistic Roles and Action

**DOI:** 10.1155/2015/384750

**Published:** 2015-09-07

**Authors:** Syed Suhail Andrabi, Suhel Parvez, Heena Tabassum

**Affiliations:** ^1^Department of Medical Elementology and Toxicology, Jamia Hamdard (Hamdard University), New Delhi 110062, India; ^2^Department of Biochemistry, Jamia Hamdard (Hamdard University), New Delhi 110062, India

## Abstract

Stroke is one of the most devastating neurological disabilities and brain's vulnerability towards it proves to be fatal and socio-economic loss of millions of people worldwide. Ischemic stroke remains at the center stage of it, because of its prevalence amongst the several other types attacking the brain. The various cascades of events that have been associated with stroke involve oxidative stress, excitotoxicity, mitochondrial dysfunction, upregulation of Ca^2+^ level, and so forth. Melatonin is a neurohormone secreted by pineal and extra pineal tissues responsible for various physiological processes like sleep and mood behaviour. Melatonin has been implicated in various neurological diseases because of its antioxidative, antiapoptotic, and anti-inflammatory properties. We have previously reviewed the neuroprotective effect of melatonin in various models of brain injury like traumatic brain injury and spinal cord injury. In this review, we have put together the various causes and consequence of stroke and protective role of melatonin in ischemic stroke.

## 1. Introduction

The brain is a highly active metabolic and complex organ of our body that performs important functions, thus, making it highly susceptible to different assaults. Any disruption in the normal functioning of the brain can lead to loss of homeostasis that can have devastating implication on whole body. Stroke leads to long-term severe disability and death [1]. There are many types of strokes like ischemic stroke, hemorrhagic stroke, and transient ischemic stroke but ischemic stroke constitutes 85% of all stroke cases which is the second leading cause of death worldwide [2, 3]. However, no effective treatment has been found to prevent the brain damage in such cases except tissue plasminogen activator with narrow therapeutic window [4–6] and there is an unmet need to develop therapeutics for neuroprotection from ischemic stroke [7]. Stroke is a broad term that refers to a range of abnormalities that are caused by occlusion or haemorrhage of one of the main arteries supplying blood to brain tissues [8]. One of the major causes of disability in ischemic stroke is the curtailment of cerebral blood flow (CBF) to a critical threshold that propagates brain damage [9]. Focal cerebral ischemia involves reduction in CBF to a specific vascular territory, usually encountered clinically due to thrombotic, hemorrhagic, or embolic strokes [10]. Within minutes of a focal ischemic stroke taking place, the core of brain tissue exposed to the most dramatic blood flow reduction is fatally injured and subsequently undergoes necrotic cell death and is called ischemia core [11]. Deprivation of oxygen by stroke is a major cause of severe neurological disability [12]. This core region is surrounded by a zone of less severely affected tissue which is rendered functionally silent by reduced blood flow but remains metabolically active [13]. This surrounding region known as the ischemic penumbra may comprise as much as half of the total lesion volume during the initial phase of ischemia and represents the region in which there is opportunity for recovery via post-stroke therapy [14]. The majority of strokes are a result of focal ischemia and one of the major blood vessels affected is the middle cerebral artery (MCA) [15]. Another type of stroke, global cerebral ischemia, involves a reduction or absence of CBF to the entire brain, situations usually encountered in severe hypotension or acute cardiac arrest. In all cases, the stroke ultimately involves dysfunction or death of brain cells, giving rise to cerebral infraction. Ischemic stroke leads to neurological deficits, cognitive impairment, and sensory impairment or in severe cases suicidal ideation [16]. This review will explore the role of reactive oxygen species (ROS), excitotoxicity, apoptosis, and current pharmacological interventions by melatonin in ischemic stroke.

## 2. ROS and Ischemic Stroke

Oxidative reactions are essential biological reactions necessary for producing high energy compounds which fuel cellular metabolic processes [17]. These processes include transfer of electrons and can generate by-products known as free radicals [18]. Brain cells have very low capacity to attenuate the effects of oxidative stress hence highly susceptible to oxidative damage involved in pathogenesis of various neurodegenerative diseases [19]. The brain derives its energy almost exclusively from oxidative metabolism in mitochondria respiratory chain that produces ROS by electron transport chain complexes in mitochondria [20]. ROS formation also takes place by degradation of free fatty acids by phospholipase A2 into arachidonic acid and subsequent oxidation of arachidonic acid by cyclooxygenase and lipoxygenase [21]. As a part of host immune system, NADPH oxidase activity in macrophages, neutrophils, and microglia also contributes to ROS production that is detrimental for the brain cells [22]. This overload of free radicals includes hydroxyl radicals (^∙^OH), superoxide (O_2_^∙−^), hydrogen peroxide (H_2_O_2_), nitric oxide (NO), and peroxynitrite (OONO-). These free radicals promote macromolecule damage such as DNA, lipids, proteins, and carbohydrates oxidation, blood brain barrier (BBB) breakdown, and microglial infiltration into the ischemic territory [23]. This production of ROS can act as intracellular signalling molecule for various destructive pathways which include apoptotic pathway. Once activated, it leads to release of apoptotic factors such as cytochrome c and apoptosis-inducing factor (AIF), ultimately leading to neuronal death [24]. In ischemia/reperfusion, the production of ROS is particularly significant during reperfusion phase that is the hallmark in the pathogenesis of cerebral ischemia [25].

## 3. Excitotoxicity

Excitotoxicity, a type of neurotoxicity, occurs when there is excessive release of neurotransmitter like glutamate for prolonged time. Glutamate is the major excitory neurotransmitter responsible for neuronal growth, axon guidance, brain development, maturation, and synaptic plasticity in health and disease [26]. Glutamate acts through three families of receptors, a-amino-3-hydroxy-5-methyl-4-isoxazole-propionic acid (AMPA), N-methyl-D-aspartate (NMDA), and kainate receptors. Out of these three, NMDA receptor is widely implicated in ischemic stroke [27]. The sequence of excitotoxicity starts with the release of excessive glutamate in the extracellular space. Excessive release of glutamate plays a prominent role in various nervous system disorders like brain trauma and ischemic injury and other neurodegenerative diseases [28]. Glutamate excitotoxicity leads to overloading of Ca^2+^ through NMDA receptor leading to activation of poly (ADP-ribose) polymerase-1 (PARP-1) and formation of poly (ADP-ribose) (PAR) polymer. PAR polymer is highly toxic to cells, killing them by sending death signals through AIF [29]. Glutamate induced overstimulation of NMDA receptor leads to increase in levels of intracellular Ca^2+^ [26]. Calcium is one of the most important signalling molecules in cell biology and maintenance of Ca^2+^ is crucial for the normal functioning of cell. In pathological conditions including ischemia/reperfusion, mitochondria accumulate significant amount of Ca^2+^ via mitochondrial calcium uniporter (MCU) from cytosol [30]. Influx of excessive Ca^2+^ into mitochondrial matrix propagates disruption of normal bioenergetic, mitochondrial ROS and increase in mitochondrial membrane permeability [31]. Excessive release of glutamate induced excitotoxicity leads to progressive neuronal death in cerebral ischemia through mitochondrial impairment and functional collapse [32].

## 4. Mitochondria and Ischemic Stroke

**Figure 1:**
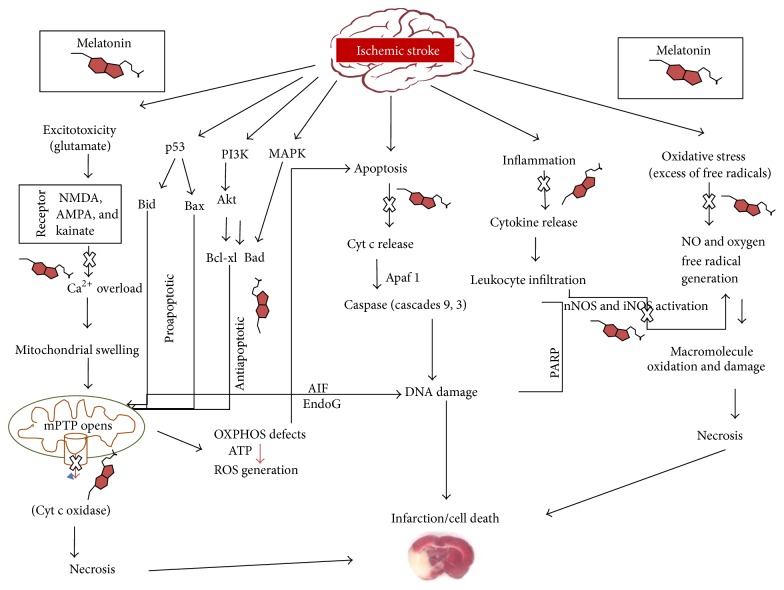
The flowchart shows a complex webbed array of events involved in the pathogenesis of cerebral ischemic injury and the role played by melatonin as neuroprotectant. Stroke onset triggers a chain of mechanisms, including activation of glutamate receptors, calcium overload with subsequent activation of apoptosis, and toxic radical release. Induction of mitochondrial permeability transition by opening of the permeability transition pore (mPTP) dissipates the mitochondrial membrane potential. These events result in cessation of electron transport and ATP formation, mitochondrial swelling, and permeabilization of the outer mitochondrial membrane, allowing the efflux of several proapoptotic molecules, including cytochrome *c* and apoptosis-inducing factor (AIF). In turn, cytochrome *c* and AIF activate a series of downstream effectors that eventually lead to the fragmentation of nuclear DNA resulting in cellular death. The cell may be also destructed by apoptosis or necrosis in case of mPTP opening and PARP activation. Triggers such as inflammation initiate the “extrinsic” pathway to programmed cell death. Conversely, calcium overload and oxygen free radicals appear to exert their effect predominantly at the mitochondrial level via the “intrinsic” pathway. In addition, crossover activation between the “extrinsic” and “intrinsic” pathway may take place through proapoptotic intermediates such as the BID protein. AIF, ATP, adenosine triphosphate; BAK; BAX, Bcl_2_-associated × protein; Bcl_2_, B-cell lymphoma 2 protein family; Bcl-X_*L*_, B-cell lymphoma-extra-large; and BID, p53 tumor suppressor protein. The ischemia-induced inflammation then further maintains these processes via cytokine release and iNOS activation leading to oxidative stress and necrosis. Melatonin limits the extent of ischemic brain injury by interacting at multisteps of the ischemic cascade. The cross indicates the interference by melatonin.

Mitochondria, a cellular powerhouse, carry out oxidative phosphorylation and generation of energy for cell. Besides being powerhouse of cell, mitochondria act as death centre by releasing several kinds of death factors like cytochrome c and AIF [43]. Once these factors are released from mitochondria, they can induce Caspase dependent (cytochrome c) and Caspase independent (AIF) cell death pathways [44]. This regulation of cell death is an important aspect of cell survival [45]. However, in cell stress, brain injury, trauma, and ischemic cell death become unregulated and lead to neurodegeneration and stroke [46]. Mitochondrial membrane contains a multicomponent protein channel composed of voltage dependent anionic channel (VDAC) in the outer membrane and adenine-nucleotide translocator (ANT) in the inner membrane [47]. Cyclophilin D (CypD), a matrix protein, and other proteins like pro- and antiapoptotic proteins primarily regulate the formation of the channel known as mitochondrial permeability transition pore (mPTP) [48, 49]. Bcl-2 associated proteins contain various antiapoptotic proteins (bcl-2, bcl-xl) and proapoptotic proteins (bax, bak) which are prime regulators of apoptosis and necrotic death [50–52]. These proapoptotic proteins play an essential role in the release of cytochrome c in cytosol through opening of mPTP [53]. During mitochondrial dysfunction, ROS/RNS and elevated Ca^2+^ levels lead to opening of mitochondrial permeability transition pore [54]. Mitochondrial overload of Ca^2+^ concentration induces mPTP opening which leads to inhibition of ATP synthesis, production of ROS, release of cytochrome c, and cell death via both apoptosis and necrosis (Figure 1) [55–57].

### 4.1. Caspase Dependent Apoptosis

Cascade of reactions lead to the opening of mPTP causing the release of proapoptotic proteins like cytochrome c into the cytosol from intermembrane space of mitochondria [58]. Once this component of electron transport chain is released into cytosol, it forms “apoptosome” by activating Apaf-1 and pro-Caspase-9. Activation of pro-Caspase-9 leads to formation of Caspase-9 which in turn activates Caspase-3 [59]. Caspase-3 has been identified as key mediator for apoptosis in animal models of ischemic stroke [60, 61]. Apoptotic protein Caspase-3 cleaves various substrate proteins such as DNA repair enzyme PARP-1. PARP-1 inactivation leads to DNA damage ultimately resulting in cell death [62].

### 4.2. Caspase Independent Apoptosis

Mitochondria can also induce apoptosis through Caspase independent pathway by releasing death proteins/factors into the cytosol. One of the best studied cell death pathways of Caspase independent proteins is release of AIF [63]. In ischemic stroke, permeability of outer mitochondrial membrane leads to the release of AIF in cytosol by proteolysis through calcium dependent calpains and calcium independent cathepsins [64]. In cytosol, AIF interacts with cyclophilin A and their translocation into nucleus leads to DNA degradation and cell death [65]. It has been well established that AIF dependent neuronal death plays a key role in acute brain injury model system of cerebral ischemia, traumatic brain injury, and epileptic seizures [66].

### 4.3. Parthanatos: A New Form of Cell Death in Stroke

Parthanatos, a new form of death mediated by PARP-1, activated product PAR polymer, known as* Par,* and* Thanatos,* a Greek word meaning “death” [67]. Oxidative stress induced free radical generation causes disruption of macromolecules like proteins, lipids, and DNA. Excessive synthesis of NO reacts to O_2_^∙−^ forms OONO- which causes DNA damage and leads to activation of DNA repairing enzyme PARP-1 [68]. Overactivation of PARP-1 causes cell death by consumption of excessive NAD^+^ or formation of toxic PAR polymer. Depletion of NAD^+^ causes ATP depletion and drop in cellular energy subsequently leading to cell demise [69]. PARP-1 also induces release of AIF from mitochondria leading to apoptosis [70]. In cell stress, DNA damage overactivates the nuclear enzyme PARP-1 producing toxic PAR polymer leading to neuronal injury via AIF release from mitochondria [71]. Inhibition of PAR polymer mediated death signalling may offer new therapeutic strategies to prevent cell injury in stroke.

### 4.4. Inflammation and Stroke

After primary events of stroke induced immunogenic cascade and immune modulatory activated inflammatory signalling contribute to ischemic damage of brain [72, 73]. There is large body evidence that ischemia involves the activation of various immune cells like monocytes, microglia, and astrocytes that exacerbates that long-term brain injury [74–76]. This recruitment of immune cells leads to the activation of various other cascades and secretion of cytokines and chemokines like IL-1, IL-6, IL-8, IL-10, and TNF-*α* modulates various inflammatory pathways in ischemia [77, 78]. Ischemia-induced BBB disruption causes infiltration of leukocytes into brain resulting in neuronal cell death [79]. Activated microglia release various inflammatory molecules, ROS and nitric oxide, that are detrimental for brain cells [80, 81].

### 4.5. Endogenous Neuroprotectants in Stroke

Endogenously occurring compounds are being implicated in various diseases like cancer and neurodegenerative diseases for their chemopreventive and neuroprotective properties. Endogenously occurring bioactive compounds such as estrogens [82, 83] and progesterone [84, 85] have been widely studied for their neuroprotective role in stroke. Among these endogenous compounds, melatonin represents one of the extensively studied compounds because of its pleiotropic neuroprotective effect in stroke (Tables 1, 2, and 3).

## 5. Melatonin

Melatonin (N-acetyl-5-methoxytrptamine) is a natural hormone secreted by pineal gland and extra pineal tissues and others such as retina, gut, bone marrow, kidney, astrocytes, platelets, and glia cells and has been used in therapies from decades in various diseases for its antioxidative and antiapoptotic properties [85–87]. Melatonin is an ideal neuroprotective agent as it readily crosses the BBB and lacks toxicity in comparison to other neuroprotectants and has been widely used for dietary supplement in various countries [88].

### 5.1. Biology and Pharmacology of Melatonin

Melatonin is formed from amino acid tryptophan by pinealocytes and rate of its synthesis depends on the activity of two enzymes, serotonin N-acetyltransferase and tryptophan hydroxylase (TPH) [89]. Tryptophan is converted into 5-hydroxytryptophan which in turn forms serotonin with the help of an enzyme aromatic amino acid decarboxylase. Serotonin is then converted into N-acetylserotonin which is the main step in the formation of melatonin. Finally, N-serotonin is converted into melatonin by another enzyme, Hydroxyindole O-methyltransferase. Out of the two, serotonin N-acetyltransferase is the main regulatory enzyme that plays pivotal role in the biosynthetic pathway of melatonin [90, 91]. Metabolism of melatonin usually takes place in liver by cytochrome P_450_ and is converted to 6-hydroxymelatonin. 6-Hydroxymelatonin is then excreted through urine in the conjugated form with sulfate and glucuronic acid [92]. There is evidence supporting the role of melatonin in regulation of various physiological functions like modulation of sleep, mood, behavior, anti-inflammatory activities, radical scavenging, immunomodulatory activities, antiangiogenic activity, and anticarcinogenic properties, and so forth [93–97]. Melatonin receptors are widely distributed in CNS as well as in the peripheral organs which acts as a lipophilic and hydrophilic molecule and able to pass the morphophysiological barriers such as the BBB [98]. Melatonin activity is mediated by the specific receptors in cellular membranes by two high affinity melatonin receptors, MT1 and MT2, which belong to the seven-transmembrane G protein-coupled receptor (GPCR) superfamily, and through nuclear receptors RZR/ROR [99]. These melatonin receptors are primarily found in the endogenous circadian master clock suprachiasmatic nuclei (SCN), located in the hypothalamus of the mammalian cells, being localized primarily to neuronal elements, and in many other organs as well, coordinate the synthesis of melatonin in the pineal gland, and also participate in several neuroendocrine and physiological processes [100].

### 5.2. Neuroprotective Action of Melatonin

Amphiphilic melatonin being a potent antioxidative and antiapoptotic agent has been used clinically in various CNS disorders [101]. It has been used in various neurodegenerative diseases like Alzheimer's, Parkinson, and stroke due to its property of inhibiting apoptotic pathways and by activating survival pathways due to its protective properties [102]. Melatonin has conferred a cerebral-protective effect, as shown by reduced infarct volume, lowered brain edema, and increased neurological scores by inducing upregulation of SIRT1 which is also associated with an increase in the antiapoptotic factor, Bcl2, and a reduction in the proapoptotic factor, Bax [103]. Melatonin (5 mg/kg) pretreatment* intraperitoneally* has diminished the increased expression of Nox2 and Nox4, reduced ROS levels, and inhibited cell apoptosis which may contribute to its antioxidant and antiapoptotic effects during brain ischemia reperfusion [38]. Mitochondrial apoptotic protein cytochrome c release was directly inhibited by melatonin (10 mg/kg b.wt.) in the model of ischemic injury [33]. Melatonin given in chronic dose has shown to redeem both impaired adult neurogenesis and the decreased density of hippocampal granule cells and reduced synaptic inhibition in Ts65Dn mouse (TS) by increasing the density and/or activity of glutamatergic synapses in the hippocampus leading to recovery of hippocampal LTP in trisomic animals. These results show that melatonin possesses cognitive-enhancing effects as seen in adult TS mice that could be mediated by the normalization of their electrophysiological and neuromorphological abnormalities. This evidence point out that melatonin represents an effective treatment in retarding the progression of Down's syndrome neuropathology [104]. Melatonin and its metabolites have been able to modulate the oxidative stress by reducing ROS, MDA, and NO and restored the GSH and SOD level in various ischemic studies [105, 106].

## 6. Neuroprotective Mechanisms of Melatonin in Ischemic Stroke

### 6.1. Reduced Oxidative Damage

It has been well documented that oxidative stress is involved in ischemic injury especially after reperfusion. It has shown that melatonin is effective antioxidant in various* in vitro* and* in vivo* models of neurodegenerative diseases not only by scavenging free radicals but also by increasing the gene expression of antioxidative enzymes like GPx, GR, and SOD [107]. Dihydroethidium (DHE) fluorescence study using a live-animal imaging system (IVIS) showed that melatonin attenuated the free radical production via MT2 receptor in mice model of ischemia [108].

### 6.2. Antiapoptotic Activity of Melatonin

Mitochondria are the prime target for melatonin in neurodegenerative diseases as it maintains mitochondrial homeostasis. Melatonin is known for its ability to inhibit release of cytochrome c from Ca^2+^ mediated mitochondria [109]. Mitochondria membrane potential is crucial factor for the maintenance of cellular bioenergetic homeostasis and its dissipation leads to formation of mPTP in stroke. Melatonin has increased the expression of antiapoptotic proteins like bcl-2 through SIRT1 pathways in mice model of cerebral ischemia. It has also decreased the expression of apoptotic factor Bax [33, 38, 103–110].

### 6.3. Inhibition of Mitochondrial Permeability Transition Pore (mPTP)

Inhibition of mPTP remains one of the prime targets in neurodegenerative diseases to block the release of death factors into cytosol [111]. It has been widely accepted that opening of mPTP takes place in stroke due to multiple stress factors, oxidative stress, and Ca^2+^ stress in mitochondria [112]. Stroke induced infarction has been seen greatly reduced in cyclophilin D deficient mice of MCAO [113]. It has been revealed by patch clamp electrophysiology that minute concentration of melatonin (250 *μ*M) directly inhibits the mPTP by interacting with channel directly [42]. Melatonin seems to maintain the mitochondrial membrane potential in various models of PCNs and PSNs that indirectly inhibits the formation of mPTP [114].

### 6.4. Regulation of Ca^2+^ Level

Excitotoxicity is one of the major events in stroke mediated via glutamate induced NMDA receptor that leads to elevation of mitochondrial level of Ca^2+^ [115]. Studies have shown that excessive Ca^2+^ leads to release of proapoptotic factor by opening of mPTP in pathological conditions [116].* In vitro* and* in vivo* studies have revealed that melatonin has perpetuated the calcium buffering proteins, parvalbumin and hippocalcin, in hippocampal cells and male Sprague-Dawley rats to attenuate the ischemic injury [117]. Stroke induced Ca^2+^ level was supposed to be reduced through inhibition of acid sensing ion channel 1a (ASIC1a) in MCAO model of stroke by melatonin [36].

### 6.5. Anti-Inflammatory Role of Melatonin in Stroke

After ischemic injury, an immune response is initiated that leads to production of proinflammatory cytokines and recruitment of various inflammatory cells like neutrophils, T-cells, macrophage, and monocytes that exacerbate the ischemic injury [118]. Melatonin regulates NO level, proinflammatory cytokines, and various enzymes like COX2 and iNOS in various neurodegenerative diseases [119]. Melatonin attenuates ischemic damage by reducing the infiltration of inflammatory cell leukocytes and microglia through MT2 receptor as shown by gp91^phos^ staining in CI/R model of mice [120]. Melatonin has mediated its anti-inflammatory effect by attenuating the glial fibrillary acidic protein level in rat model of cerebral ischemia. It has been shown that melatonin also reduces the activation of microglia and infiltration of monocytes. [121]. Melatonin successfully reduced the microglia production of NO by decreasing the iNOS level in ischemic model of rat [106].

### 6.6. Regulation of PI3K/Akt Pathway

It is well documented that PI3K/Akt pathway plays important role in neuronal death and survival [122]. Melatonin has shown positive modulation through this pathway in various neurodegenerative disorders [123]. PI3K/Akt pathway is the important survival pathway in neurons by targeting antiapoptotic factors like Bcl-2 protein family activated by Akt. These antiapoptotic proteins inhibit apoptosis by subjugating the apoptotic pathways in mitochondria. Studies have shown that activating the Akt leads to neuroprotection by inhibiting apoptosis in various models of stroke [124, 125]. Melatonin has strongly inhibited autophagy and stimulated the PI3K/Akt prosurvival pathway in MCAO model of ischemia [126]. Melatonin can target PI3K/Akt pathway, mTOR, or the forkhead transcription factor pAFX and also restore JNK1/2 and ERK 1/2 phosphorylated levels, thereby preventing the proapoptotic actions of the dephosphorylated proteins [127]. Reports are available on the role of phosphatidyl inositol-3 kinase/Akt signaling in acute melatonin-induced neuroprotection, while ERK-1/-2 and/or JNK-1/-2 rather appear to be involved in melatonin's long-term effects [128].

### 6.7. Regulation of MAP Kinase Pathway

Few others suggest that protection from cerebral ischemic injury was attributed to the maintenance of signalling via the mitogen activated protein kinase pathway, leading to the prevention of Bad dephosphorylation. MAP kinase pathway has been involved in various cellular processes like cell differentiation, growth, death, and cell survival. Bcl-xl, an apoptotic protein, is regulated in mitochondria through phosphorylation of p38 MAPK imparts neuroprotection in MCAO model of mice [128]. Phosphorylation of ERK1/2 via activated Raf and MEK by various growth factors leads to phosphorylation of antiapoptotic protein, Bad, by phosphorylated ribosomal S6 kinase (p90RSK). Melatonin has alleviated the neuronal death in cerebral ischemia by activating signalling cascade of Raf/MEK/ERK/p90RSK in ischemic model [37, 129].

### 6.8. Regulation of Endothelin-1 Pathway

Several other mechanisms behind melatonin's neuroprotective action have been proposed. A study shows that melatonin, both when prophylactically administered and when acutely applied, is a powerful endothelin converting enzyme-1 (ECE-1) inhibitor. In humans, endothelin-1 is implicated in the evolution of arterial hypertension, stimulates platelet aggregation, and also reinforces the formation of ROS. Activation of the endothelin-1 pathway is therefore considered to confer an increased risk of stroke and MEL as its inhibitor have been proposed as a treatment for vascular disease [130].

#### 6.8.1. Role of Nrf2

Investigators have suggested that melatonin as a neuroprotectant in cerebral ischemia may involve mechanisms like reduction in ROS production, and activation of the nuclear factor-erythroid 2-related factor 2 (Nrf2), which is a master regulator of endogenous antioxidant defenses followed by overexpression of phase II enzymes such as heme oxygenase-1, which has a potent antioxidant and anti-inflammatory effect [131].

#### 6.8.2. Receptor Proteins Involved in Melatonin Neuroprotection

Melatonin acts via high affinity G-protein-coupled melatonin receptors, MT1 and MT2, which have been identified* in vitro* autoradiography and conventional binding assays, also cloned and characterized in mammals. The specific receptors have been found in human brain regions like medial preoptic area, anterior hypothalamus, paraventricular and anteroventral thalamic nuclei, hippocampus, cerebral and cerebellar cortex, and retina. The available data indicates that the melatonin receptor is associated with membrane and is also linked to secondary messengers such as cAMP, cGMP, diacylglycerol, arachidonic acid, IP3, and inorganic calcium. Melatonin also regulates the third messengers, namely, the phosphorylation of CREB and expression of c-Fos [132]. Data suggest that melatonin exhibits a protective effect against ischemic stroke in mice model which is MT2 melatonin receptor-associated. Studies have indicated that melatonin elicits its neuroprotective effect in ischemic stroke through MT2 receptor as revealed by using MT2 receptor antagonist luzindole [108]. Also, activation of the MT2 melatonin receptor in the hippocampal region with melatonin treatment shown by immunoreactive responses may be involved in its neuroprotective action against transient cerebral ischemic damage [133]. But few studies using knock-out mice show contradictory results that both MT1 and MT2 receptors are not necessary for neuroprotection by MEL against ischemia [134].

## 7. Future Perspectives of Melatonin

Melatonin, a neurohormone, has been found effective in various animal models of brain injury. Several research studies are being done on assessing protective role of melatonin in humans. Melatonin, a potent antiapoptotic and antioxidative neuroprotectant with no serious toxicity, raises hopes that it might be used for humans for stroke treatment. The bulk of studies published have used pharmacological interventions against ROS production and apoptosis. There is need to explore different mechanisms of melatonin in neuroprotection of different models of brain injury that will be more effective at endogenous levels of body that might be important especially at later stage of age when melatonin level is attenuated. There is need to design more efficient clinical trials to explore the clinical aspect of protective role of melatonin in detail against various neurodegenerative diseases.

## Figures and Tables

**Table 1:** Effect of melatonin on ischemic model of rat.

Dose	Duration	Effect/result	References
5 mg/kg b.w, i.p	30 min before MCAO	↓ROS, ↓NOX2, and ↓NOX4 expression, ↓TUNEL positive cells	[33]

5 mg/kg b.w, i.p	30 min before and 60, 120 min after occlusion	↓nitrite level, ↓MDA, and ↓Ca^2+^	[34]

5 mg/kg b.w, i.p	At 90 min of reperfusion	↑PSD-95, ↑GAP-45, and ↑MMP-9	[35]

5 mg/kg b.w, i.p	Prior to MCAO	↑parvalbumin and ↑hippocalcin ↓Ca^2+^	[36]

5 mg/kg b.w, i.p	Prior to MCAO	↓phosphorylation of Raf-1, MEK1/2, ERK 1/2, and ↓TUNEL positive cells	[37]

**Table 2:** Effect of melatonin in mice model of ischemic stroke.

Dose	Duration	Effect/result	References
10 mg/kg b.w, i.p	Twice at ischemia reperfusion	↑SIRT1, ↑BCL-2, and ↓Bax. ↓mitochondrial membrane potential, ↑mitochondrial complex I, ↑mitochondrial cytochrome c, and ↓cytoplasmic cytochrome c level	[38]

5 mg/kg b.w, i.v	Upon reperfusion	↑TIMP expression, ↑PAI activity, ↓uPA activity, and ↓MMP-9	[39]

4 mg/kg b.w, oral	After 24 hr ischemia for 29 days through drinking water	↑neuronal survival, ↑rotarod, ↑grip strength, and ↓anxiety and ↓hyperactivity	[40]

**Table 3:** Effect of melatonin on OGD model.

Dose	Duration	Effect/result	References
10 and 100 nM	Before OGD for 24 hrs	↑activation of Akt, ↓phosphorylation of JNK	[41]

10^−5^, 10^−7^, 10^−9^	After reperfusion	↓mitochondrial membrane potential, ↓cytoplasmic cytochrome c and ↓Caspase-3 and ↓DNA damage	[39]

100 to 250 *µ*M	At time of OGD and oxygen-glucose resupply	↓mPTP, ↓mitochondrial depolarization, ↓Ca^2+^ level, ↓Caspase-3 activation, ↓DNA fragmentation, and ↓cytochrome c release	[42]
